# Uterine Carcinosarcoma in a Patient with Didelphys Uterus

**DOI:** 10.1155/2013/401962

**Published:** 2013-03-07

**Authors:** C. Iavazzo, F. Kokka, A. Sahdev, N. Singh, K. Reynolds

**Affiliations:** Gynaecological Department, St Bartholomews Hospital, London EC1A 7BE, UK

## Abstract

*Background*. Didelphys uterus is a noncommon finding in women. Till now, few cases with benign mesenchymal tumors in patients with didelphys uterus are described. We present a case of a patient with carcinosarcoma arising in a didelphys uterus. *Case*. A 73-year-old patient presented with profuse watery postmenopausal bleeding. On examination under anesthesia, left and right cervixes were identified. Tumor extended from the left cervix into the lower third of the vagina and was adherent to the right vaginal sidewall. There was no evidence of parametrial extension. Tissue was sent for biopsy which revealed high-grade uterine carcinosarcoma. Two uterine fundi and two vaginas in keeping with uterine didelphys were identified on imaging. The patient underwent vaginal excision of the protruding tumor measuring 8 × 6 cm with harmonic scalpel followed by total abdominal hysterectomy and bilateral salpingooophorectomy. Although a number of pelvic and paraaortic lymph nodes were also identified on imaging, she was not planned for lymphadenectomy after MDT (multidisciplinary team) discussion because of her comorbidities. The final histology confirmed the diagnosis. *Conclusion*. According to our knowledge, this is the second case of carcinosarcoma arising in didelphys uterus in the world literature.

## 1. Introduction

Persistent Müllerian duct syndrome presenting as didelphys uterus is a very rare anomaly which is estimated to occur in 1/3,000 women [1]. However, it might go undetected in the absence of medical and reproductive complications and so the incidence may be underestimated. Fibroids are benign mesenchymal tumors which are commonly associated with cases of didelphys uterus and are present in premenopausal women [2]. However, sarcoma—the malignant mesenchymal tumor—is an extremely rare finding in a woman with a didephys uterus. We are only aware of one case previously described in the literature [3]. Moreover, very few reports of endometrial cancer arising in patients with uterine malformations could be also found in the literature [4–7]. We present a case of carcinosarcoma found in a woman with a didelphys uterus (Figure 3). A review of the literature was performed to clarify the diagnostic pathway and the management of this rare entity.

## 2. Case

A 73-year-fold patient, para 5, presented with profuse watery postmenopausal discharge and frank red bleeding per vagina for 2 weeks. On examination under anesthesia, left and right cervixes were identified. Tumor extended from the left cervix into the lower third of the vagina and was adherent to the right vaginal sidewall. The uterus was otherwise mobile, and there was no evidence of parametrial extension. Tissue was sent for biopsy which revealed high-grade uterine carcinosarcoma.

**Figure 1:**
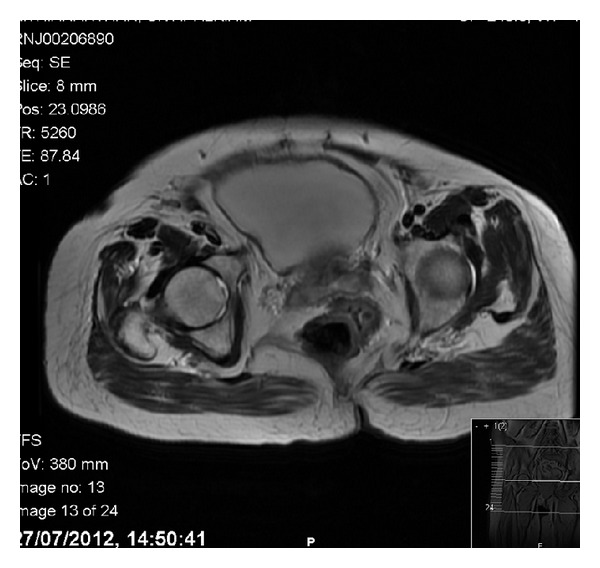
Coronal image of the tumor and the didelphys uterus.

**Figure 2:**
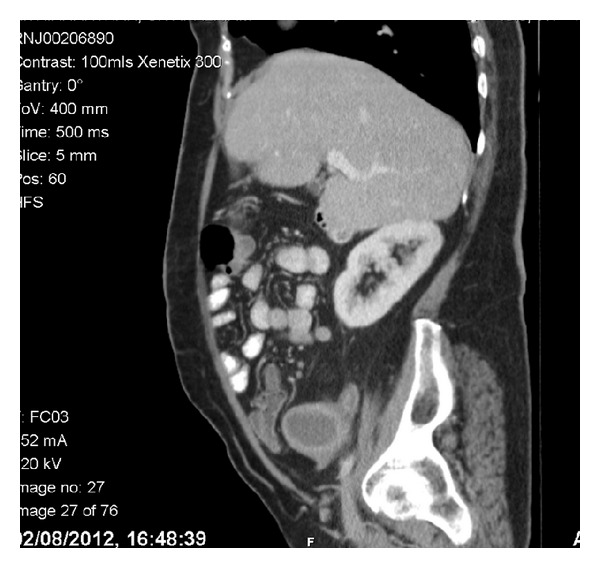
Sagittal image of the tumor and the didelphys uterus.

**Figure 3:**
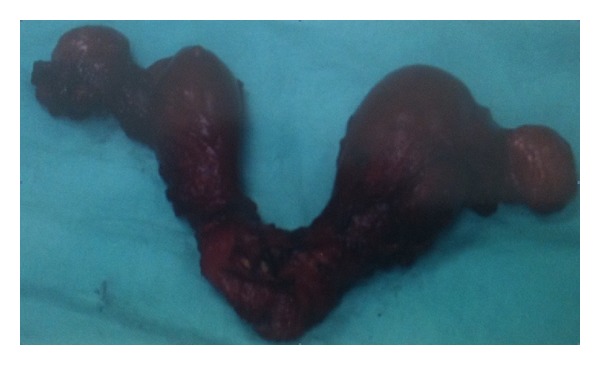
A posterior view of a didelphys uterus from a 73-year-old woman with uterine carcinosarcoma is shown.

After contrast-enhanced computed tomography (CT) of the chest, abdomen and pelvis showed two uterine fundi and two vaginas in keeping with uterine didelphys (Figure 1). Small volume lymphadenopathy was present with 1 cm precaval lymph node, 1 cm right common iliac lymph node and 9 mm rounded right external iliac lymph node. No lung disease or significant bony lesions were found. Magnetic resonance imaging (MRI) confirmed two uterine fundi, cervices, and vaginas (Figure 2). The right uterine fundus had a distended endometrial cavity measuring 2.9 cm. Multifocal endometrial soft tissues were present. A soft tissue mass with projections was present at the fundus and in the distal endometrial canal extending into right endocervical canal and invading the anterior cervical stroma. The soft tissue mass extended into the upper one-third of the right vaginal cavity. No definite evidence of parametrial invasion was found, while the lower two-thirds of the vagina were normal. The left uterine fundus, cervix, and vagina were normal. 

Positron emission tomography (PET) CT demonstrated a metabolically active tumour with intense metabolic activity corresponding to local uterine disease sites seen on MRI. PET CT also detected metabolically active nodes in the left supraclavicular fossa, mediastinum, abdomen, and pelvis in keeping with metastatic disease.

Although a number of pelvic and paraaortic lymph nodes were identified on imaging, she was not planned for lymphadenectomy after MDT discussion because of her past medical history which included coronary artery bypass graft 3 years ago, hypertension, and unstable diabetes mellitus. The patient had three laparotomies as a child, the third of which resulted in a bowel resection due to anatomical duplication of her gastrointestinal tract. On examination, the patient had reduced exercise tolerance (angina at 400 yards) and also had evidence of reflux on induction of anaesthesia and respiratory wheezing. 

The patient underwent vaginal excision of the protruding tumor measuring 8 × 6 cm with harmonic scalpel followed by total abdominal hysterectomy and bilateral salpingooophorectomy which was achieved after mobilizing the uteri from the adjacent bladder and bowel which were adherent in the midline between the right and left uteri. Two separate uterine bodies were found, each with its own cervix and attached fallopian tube and ovary. The area of the tumor in the vaginal wall was marked with clips as the patient would have adjuvant treatment postoperatively. It should also be mentioned that the caecum was found double in size. Dilated small bowel loops were also found. The patient had an uneventful postoperative recovery.

The final histology confirmed the diagnosis of carcinosarcoma stage IIIb. More specifically, the vaginal tumour was a polypoid necrotic tumour consisting of malignant columnar epithelial cells within a stroma consisting of highly atypical spindle-shaped cells. The epithelial component consisted mainly of high-grade serous carcinoma and showed marked atypia with prominent mitoses. The stromal component consisted of markedly atypical spindle and stellate cells. Occasional foci consisted of smaller ovoid cells with hyperchromatic nuclei and granular chromatin. No heterologous components were seen. The features were of a high-grade carcinosarcoma. Some residual foci of vaginal epithelium and stroma were also seen. Regarding the uteri, tubes, and ovaries, the right horn of the uterus showed a carcinosarcoma with features similar to those of the vaginal tumour. Within the corpus this consisted mainly of high-grade serous adenocarcinoma with very focal sarcomatous differentiation. Psammomatous calcification was also seen. The tumour invaded into the outer half of the myometrium and was less than 1 mm from the serosal surface. Extensive lymphovascular space invasion was seen. The right cornu, right tube, and ovary also showed tumour involvement. Tumour was present at the endocervical resection margin. The right and left horns showed tumour involvement. Tumour was also seen at the parametrial margins, bilaterally. The left ovary contained a focus of calcification and foreign body giant cell reaction but no definite tumour involvement. The left horn of the uterus showed a benign endometrial polyp. The background endometrium showed foci of simple hyperplasia without atypia. Some occasional foci of adenomyosis and benign leiomyomas were also present. The cytology of the washings revealed a cellular specimen consisting of cohesive groups of atypical cells, with prominent nucleoli. The cells aggregated to form glandular structures. Background reactive mesothelial cells and histiocytes were also present. The features were of metastatic carcinoma. The final histopathology was discussed at the Gynae Oncology MDT, and palliative radiotherapy was recommended.

## 3. Discussion

Fusion uterine anomalies of various kinds are not uncommon. Such anomalies result from differing degrees of failure of fusion of the two Müllerian ducts at about 9 weeks of gestation. A congenital anomaly syndrome with didelphys uterus and ipsilateral renal agenesis was first reported in 1922 [8]. Since then, over 180 cases were reported in the literature [5].

Most of these anomalies do not reduce the female fertility, and this was demonstrated in our patient as she had five normal deliveries. However, complete duplication of the uterus and cervix may prevent descent of the fetal head in late pregnancy or obstruct labor by the nonpregnant horn, something that was mentioned in our patient's medical history.

Usually, women with a congenital uterine anomaly have an increased risk of renal anomalies and should undergo renal tract imaging. Our case was not correlated with significant renal anomalies, but she had a history of gastrointestinal tract anomalies (duplicate colon). The other finding from the genitourinary tract was that our patient had normal urethra, as well as a second “blind” urethra. 

Uterine malformations may cause a delayed diagnosis of gynecological malignancies. Imaging studies including ultrasound, 3D ultrasound, CT, and MRI could help in the diagnosis of uterine malignancies in cases of didelphys uterus [5]. We used ultrasound and MRI for the diagnosis of didelphys uterus, as well as PET scan for clarification of lymph node metastases. However, it should be mentioned that the diagnosis is based on the histological findings.

A Pubmed and Google search was conducted for internet-based resources and open access publications with the terms didelphys uterus or double uterus and cancer or sarcoma or malignancy and we identified 17 cases with endometrial cancer and another case with carcinosarcoma between the years 1952 and 2012 [3–21]. So according to, our knowledge our case is the second described in the literature with carcinosarcoma in a patient with didelphys uterus. The first case of carcinosarcoma of the uterus is related with tamoxifen use in a 72-year-old patient with a history of breast cancer [3]. The patient similarly to our case presented with a large pelvic mass, but the tumor was not recognized preoperatively. This patient died of the disease 5 months after diagnosis. So both cases presented in postmenopausal women over 70 years with rather large tumors.

The question of why the patient underwent MRI could be raised as the role of MRI is to identify patients likely to be at risk of nodal disease and thereby select patients for lymphadenectomy. CT or PETCT alone would have sufficed for the detection of distant disease, whilst local staging was surgical. However, we performed first the imaging and after that the MDM decided to avoid lymphadenectomy. 

Carcinosarcomas are also called mixed Müllerian tumors which are characterized by a typical biphasic pattern with carcinomatous and sarcomatous elements (either homologous or heterologous). In our case, no heterologous components were found. The behavior of carcinosarcomas is characterized mainly by the carcinomatous component, and so mainly they give lymph node metastases. In our case, pelvic and paraaortic lymph nodes were identified, but because of the comorbidity, a lymphadenectomy was not performed. Radiotherapy is suggested in the literature as another option for treatment of uterine didelphys malignancy [3]. In general, it is known that the overall 5-year survivals of patients with uterine carcinosarcoma receiving radiotherapy versus no irradiation were 41.5% and 33.2%, respectively. Combined adjuvant radiotherapy and chemotherapy may prolong even more the 5-year survival. 

## 4. Conclusion

Didelphys uterus is a rare finding in women. It is even more rare to be complicated with carcinosarcoma. According to our knowledge this is the second case of carcinosarcoma arising in didelphys uterus in the world literature.

## References

[1] Grimbizis GF, Camus M, Tarlatzis BC, Bontis JN, Devroey P (2001). Clinical implications of uterine malformations and hysteroscopic treatment results. *Human Reproduction Update*.

[2] Johnsrud ML (1994). Successful pregnancy outcome in uterus didelphys with leiomyoma uteri. *Acta Obstetricia et Gynecologica Scandinavica*.

[3] Kunos C, Woods C, Colussi VC, Abdul-Karim FW, Waggoner S (2011). Low-dose-rate brachytherapy for treatment of uterine didelphys malignancy. *Journal of Clinical Oncology*.

[4] Chen CY, Yen MS, Yang MJ, Wu YC (2008). Uterus didelphys with adenocarcinoma in the right cavity diagnosed by 2-dimensional sonography and magnetic resonance imaging. *Journal of Ultrasound in Medicine*.

[5] Fanfani F, Fagotti A, Restaino G, Guerriero M, Scambia G (2006). Endometrial cancer arising in both horns of didelphys uterus in a Down’s syndrome woman. *Gynecologic Oncology*.

[6] Bhalla R, Evans H, Berger L, Crow J, Deheragoda M, Taper Y (2005). A uterus didelphys bicollis, with endometrial cancer in both uteruses. *Journal of Obstetrics and Gynaecology*.

[7] Purslow CE (1922). A case of unilateral haematocopos, haematometra and haematosalpinx. *BJOG: An International Journal of Obstetrics & Gynaecology*.

[8] Fealy J, Nelson JH (1957). Adenocarcinoma in one-half of a uterus didelphys. *The Medical Annals of the District of Columbia*.

[9] Braun RD (1970). Uterus didelphys and endometrial carcinoma. A case report. *Obstetrics and Gynecology*.

[10] Grant KB, Sedlacek RL (1970). Uterus didelphys with adenocarcinoma in one fundus—a case report. *Journal of the Iowa Medical Society*.

[11] Anneberg AD (1971). Double vagina with double uterus (didelphys) containing endometrial adenocarcinoma. Report of a case. *Journal of the Iowa Medical Society*.

[12] Eichner E, Simak KA (1981). Uterus didelphys unicollis with adenocarcinoma in one horn and atypical endometrial hyperplasia in the other: case report. *American Journal of Obstetrics and Gynecology*.

[13] Suprasert P, Khunamornpong S (2010). Carcinosarcoma arising in uterine didelphys after tamoxifen therapy for breast cancer: a case report. *Journal of the Medical Association of Thailand*.

[14] Thomas AG, Deligdisch L, Goldstein M (1988). Endometrial carcinoma with uterus didelphys. *Mount Sinai Journal of Medicine*.

[15] Woods MS, Sheppard RG, Hardman DA, Woods HJ (1992). Congenital genitourinary anomalies: is there a predilection for multiple primary malignant neoplasms?. *Cancer*.

[16] Chen FP, Ng KK (2006). Term pregnancy at the site of atresia following vaginal canalization in a case of uterus didelphys with hemivaginal atresia and ipsilateral renal agenesis. *Taiwanese Journal of Obstetrics and Gynecology*.

[17] Pojman DV, Taxy JB (1995). Images in clinical medicine. Double uterus with adenocarcinoma. *The New England Journal of Medicine*.

[18] Kosinski A, Dini M (1994). Endometrial cancer in a double uterus: a report of two cases. *The Journal of Reproductive Medicine*.

[19] Vial’tsev NV, Sokolova NV, Pronin AG (1991). Development of endometrial cancer in one half of a double uterus. *Arkhiv Patologii*.

[20] Tsukahara Y, Fukamatsu Y, Tomita K, Shiozawa T, Iinuma H, Fukuta T (1990). Endometrial carcinoma arising from a double uterus. *Gynecologic and Obstetric Investigation*.

[21] van Assen FJ (1970). An exceptional case of endometrial carcinoma or a double tumour of the uterus?. *Nederlandsch Tijdschrift voor Verloskunde en Gynaecologie*.

